# Culturally adapted mental healthcare: evidence, problems and recommendations

**DOI:** 10.1192/pb.bp.115.050872

**Published:** 2015-08

**Authors:** Sokratis Dinos

**Affiliations:** 1 BPP University, London, UK

## Abstract

Evidence suggests disparities in the prevalence of mental health problems and access to mental healthcare for a number of minority groups. The main response from mental health services falls into two related categories: (a) cultural adaptations of existing evidence-based interventions (EBIs) and/or (b) cultural competence of mental health professionals. This editorial looks at the evidence on culturally adapted EBIs and argues that although such interventions can be effective, they also carry the risk of alienating members of the groups they are aimed at. Recommendations focus on identifying issues that pertain to being from a racial minority and/or possessing other stigmatised identities that can have an impact on mental health problems, which may be overlooked by mental health services by assuming an overarching predominant cultural identity.

Evidence suggests that disparities exist in the prevalence of mental health problems and access to mental healthcare for a number of high-risk groups.^1^ Many of these groups share similar characteristics in terms of minority status (e.g. Black and minority ethnic (BME) groups) or belong in subgroups that exist within a larger racial/ethnic population (e.g. lesbian, gay, bisexual and transgender (LGBT) people, older people, refugees, asylum seekers). Findings from earlier and more recent reviews suggest that mental health services are unattractive to these diverse cultural and subcultural minority groups, who complain of stigmatisation, prejudice, unsuitable treatments and adverse experiences and as a consequence, seek care less frequently and drop out of contact more often than others.^2,3^ Anxiety about potential discrimination or inability of the services to understand the diversity of needs can cause reluctance to access healthcare, resulting in delayed treatment.

The main response from mental health services to deal with mental health problems in minority populations falls into two related categories: (a) cultural adaptations of existing evidence-based interventions (EBIs) to clients' cultures and backgrounds and/or (b) cultural competence of health professionals in terms of specialist knowledge relating to particular lifestyles and needs. It can be argued that cultural adaptations do not automatically translate into mental health professionals' cultural competence and similarly, cultural competence of health professionals does not translate to provision of suitable, culturally adapted EBIs. Although culturally adapted EBIs seem to be the right way forward, they can also be conceptually simplistic and narrow. On the other hand, cultural competence can solve many of the conceptual problems that cultural adaptations may pose but any subsequent interventions need to be evidence based.

## Cultural adaptations: do they work?

Culture is a complex concept encompassing a number of elements, including shared knowledge, language, behaviours, cognitive constructs (e.g. thoughts, schemas, beliefs, norms). Health practices such as those around food and exercise, health beliefs about aetiology, course and outcome of illness as well as health behaviours are also heavily influenced by culture.^4^ Cultural adaptations of mental health EBIs incorporate some or all of these elements with the aim of narrowing inequalities in care and reducing the higher prevalence of mental health problems in disadvantaged groups.

Meta-analyses of such interventions have produced mixed, inconclusive or positive results.^5-8^ In particular and when compared with traditional EBIs, control groups and/or other care, effect sizes have ranged from small to moderate (0.21-0.46). Interventions analysed have included individual therapy or group therapy or a mixture of both. Clinical characteristics included at-risk groups, clinical populations already diagnosed with a mental illness, and community members without a psychiatric diagnosis. There was no reference to whether services were in-patient or out-patient. Results from the meta-analyses did not differ by type of intervention, clinical characteristics, gender and ethnicity. One of the meta-analyses has also produced effect sizes for a number of different elements of culturally adapted EBIs.^7^ For example, cultural, ethnic and racial matching of individuals from BME groups with service providers was more effective than when clients were not matched with the provider or therapist (d = 0.58 *v.* d = 0.31). Furthermore, non-English and/or ethnic-specific services were more effective than non-ethnic-specific services (d = 0.49 *v.* d = 0.21).

## Culture *v.* subculture: does one size fit all?

It can be argued that overall the evidence for culturally adapted EBIs is encouraging. However, cultural adaptations may also carry the risk of alienating members of those groups they are aimed at. One of the risks is that mental health professionals and researchers often make the assumption that individuals from specific subgroups possess certain cultural characteristics^9,10^ and fail to take into account that within a larger group there are a number of subgroups with different characteristics, which may be overlooked by attaching an overarching cultural identity to them. These subgroups may also be those with the highest risk of developing mental health problems. We can refer to them as subcultural groups which exist within a larger racial population and share similar life experiences and a mutual sense of belonging.

For subcultural groups racial identity may be secondary and the primary identity may be either more in tandem with the mainstream culture where they reside or related to a different group membership. Such group membership may be related to another stigmatised identity such as ethnic minority, LGBT groups, refugees, asylum seekers, older people. Therefore, some minority groups may find themselves in membership of multiple stigmatised identities in addition to ethnicity and mental illness (e.g. sexuality, single motherhood, asylum seeker, offending, poverty). In these situations mental health professionals would need to question whether an existing culturally adapted intervention would be beneficial. Therefore, subcultural differentiation is important when both adapting EBIs and delivering a culturally competent mental healthcare, as it provides specific information that goes beyond the ethnic identity and can capture other characteristics and/or needs that are not ethnic specific.

A further risk of cultural adaptations is the cultural contexts where the original interventions were developed. In particular, the majority of EBIs have been developed with participants from majority groups (e.g. Western, White) and then have been culturally adapted and applied to minority groups residing in the context where these were developed originally. Since many cultural adaptations are developed from existing EBIs, they very often overlook the factors related to being a minority, such as racism, stigma, poverty, internalised oppression, and mainly focus on culture and mental health. For example, coping with a stigmatised identity has been found to be a crucial factor in recovering from mental illness in a number of studies. In particular, in a systematic review of published descriptions and models of personal recovery, Leamy *et al*^11^ found that recovery for ethnic minority groups involved racial discrimination, stigma of mental illness and stigma of ethnic minority identity. Therefore, recovery was not as narrowly defined as recovering from mental illness.

A good example of incorporating these issues can be found in a culturally adapted mindfulness-based stress reduction (MBSR) intervention developed by Dutton *et al*.^12^ The intervention was adapted for African American women from low socioeconomic background who were in abusive relationships and experienced post-traumatic stress disorder. Interviews and focus groups revealed that the main concerns were related to problems posed by living on a low income, lack of space to practise MBSR exercises (e.g. breathing, yoga, meditation), exposure to trauma, and also the structure of the intervention (e.g. time frames and length of sessions) in relation to childcare demands. Therefore, the adaptation involved, among other elements, shorter sessions and availability of childcare as well as a special focus on dealing with the everyday stressors of low-income existence and coping with trauma. The specificity of race did not appear to be a theme that needed to be included in the development of the intervention. However, the stigma of mental illness and the stigma of using mental health services, which has been found to be prevalent among African American populations,^13^ was also evident in the interviews and led to adaptations that involved elements to cope with the stigma of mental illness.

## Where next?

Overall, it can be argued that mental health services are going in the right direction in terms of culturally adapting EBIs to reach at-risk groups and narrow the gap of mental health inequalities. However, cultural adaptations can be beneficial if applied by health professionals who are culturally competent and have the ability to explore differing values and needs with their clients instead of assuming cultural characteristics that may be either non-existent or not predominant. Therefore, training of staff which focuses on removing prejudice and promotes cultural competence and specialist knowledge pertaining to particular lifestyles needs to go hand in hand with cultural adaptations of EBIs. Cultural competence will help mental health professionals to make an assessment focusing on each client's experience before applying cultural adaptations. Such assessments need to happen on a case by case basis and mental health professionals need to identify a number of issues before making a decision. Such issues pertain to: (a) exploring the predominant identity of the client (e.g. cultural or other), (b) identifying issues that may be related to being a racial minority (e.g. internalised racism and discrimination) and/or having a mental illness (e.g. mental illness stigma in a particular culture) and (c) exploring the client's membership in other groups that may be stigmatised or have an impact on mental health (e.g. LGBT, low socioeconomic status).

In relation to exploring a client's predominant identity, Rucker Sobczak & West^14^ suggest that the initial assessment needs to involve an understanding of whether the client subscribes to a collectivistic (e.g. seeing the self as part of a cultural group or others with collective goals) or individualistic (e.g. seeing the self as a separate entity to others with individual goals) self-identity as this can have significant outcome implications. Such assessment can also add clarity about whether there is a dominant identity and whether this is the cultural one. Similarly, assessment measures that deal with issues related to being in a minority group such as stigma and perceived racism may reveal what type of interventions may be more beneficial for, or applicable to, which individuals.^10^

Finally, it is important to stress that cultural adaptations and cultural competence have to operate along other initiatives that aim to reduce discrimination and tackle social exclusion, which have an impact on further socioeconomic disadvantage and place someone at increased risk of mental illness, and to promote outreach effort to recruit underserved clients from high-risk groups and actively target communities with higher concentration of socially excluded groups (e.g. BME communities). Needless to say, culturally adapted EBIs and cultural competence training as well as initiatives to reduce discrimination and social exclusion must be evidence based and be subject to evaluations on what works and for whom. Currently, most of the evaluation data on culturally adapted EBIs come from US studies, so future interventions and subsequent evaluations need to happen in a UK context.
